# Plant Iron Research in African Countries: Current “Hot Spots”, Approaches, and Potentialities

**DOI:** 10.3390/plants13010014

**Published:** 2023-12-19

**Authors:** Irene Murgia, Piero Morandini

**Affiliations:** Department of Environmental Science and Policy, Università degli Studi di Milano, Via Celoria 10, 20133 Milan, Italy; piero.morandini@unimi.it

**Keywords:** Africa, biofortification, crops, Fe deficiency, Fe toxicity, nanoparticles, nutrition, research dissemination, rice, sustainable agriculture

## Abstract

Plant iron (Fe) nutrition and metabolism is a fascinating and challenging research topic; understanding the role of Fe in the life cycle of plants requires knowledge of Fe chemistry and biochemistry and their impact during development. Plant Fe nutritional status is dependent on several factors, including the surrounding biotic and abiotic environments, and influences crop yield and the nutritional quality of edible parts. The relevance of plant Fe research will further increase globally, particularly for Africa, which is expected to reach 2.5 billion people by 2050. The aim of this review is to provide an updated picture of plant Fe research conducted in African countries to favor its dissemination within the scientific community. Three main research hotspots have emerged, and all of them are related to the production of plants of superior quality, i.e., development of Fe-dense crops, development of varieties resilient to Fe toxicity, and alleviation of Fe deficiency, by means of Fe nanoparticles for sustainable agriculture. An intensification of research collaborations between the African research groups and plant Fe groups worldwide would be beneficial for the progression of the identified research topics.

## 1. Introduction

Plant iron (Fe) research deals with the multifaceted strategies adopted by plants for Fe uptake from soils of various pHs [1,2], its transport, metabolism, signaling, and distribution from roots to other districts, such as leaves and seeds [3,4], and the biochemistry of Fe-requiring enzymes FeRE [5,6] and Fe complexes [7,8]. Fe homeostasis is influenced by below- and above-ground environments, including detrimental or beneficial living organisms [4,9]. A relevant branch of plant Fe research is devoted to amelioration of bioavailable Fe content in edible parts of staple crops for improving human nutrition and for combating Fe deficiency anemia [10,11,12]. These research goals require competency in genetics, agronomy, biotechnology, and both human and plant physiology, and they could benefit from the identification of novel hubs in plant nutrition [13]. The various micro- and macronutrients reciprocally influence each other in terms of their homeostasis, as has emerged, for example, between Fe and molybdenum [14] and between Fe and sulfur [15], clearly indicating that “no plant nutrient is an island”. 

Hence, research of all these aspects of plant Fe metabolism is of worldwide importance, including the African continent. Indeed, African countries can count on incredible plant biodiversity and a variety of environments, and they can contribute to expanding the number of species employed for Fe research. Moreover, several African populations experience severe Fe deficiency anemia (IDA) [16] exacerbated by malaria, which is endemic in various African countries. Fe-rich staples are therefore needed. However, Fe metabolism of some of the species which are nutritionally relevant in Africa (e.g., millets) is not investigated as intensively as other more globally relevant crops.

Unfortunately, plant Fe research prominently conducted by scientists affiliated with African research centers does not always enjoy full international visibility and dissemination. This can be ascribed to several factors, besides research funding, such as participation in international congresses, publications in relevant journals in the field, availability of scholarships, student mobility, rigor of research, etc. 

The analysis of all these aspects is beyond the scope of the present work, which instead aims to fill this gap of visibility and dissemination. For that, we focused on research conducted by scientists affiliated with African universities/research institutions and focusing on plant Fe nutrition and metabolism in recent years (2018–2023). Interestingly, three major topics of interests emerged, which are discussed in the following paragraphs.

## 2. Plant Fe Research Prominently Conducted in Africa, from 2018 to 2023

Various databases of scholarly literature are available; the Scopus database (https://www.scopus.com/home.uri, accessed on 14 September 2023) was used for the present work, as it allows user-friendly searches with multiple exclusions and/or restrictions in various fields. The query in Scopus was conducted by searching all research papers or reviews published in English from 2018 onwards, in which the term “iron” appears in the title. Only “Agricultural and Biological Sciences” and “Biochemistry, Genetics and Molecular Biology” subject areas were considered; all the other subject areas were excluded. To restrict searches to plant science, several keywords (Supplementary Figure S1) as well as various journals (Supplementary Figure S2) were used as criteria for exclusion. The search was then limited to authors affiliated with African countries. The final list of 92 publications was then manually pruned to exclude publications whose main focus was not on plant science (e.g., publications mainly on nutrition were excluded). At the end, 69 publications were retained, affiliated with 26 countries (Supplementary Figure S3). Although not retrieved by this procedure, one more publication [17] was added to the list, as it represents the beginning of a work [18] included among the 69 publications. 

About 50% of these 70 publications are focused on rice (various *Oryza* species), maize (*Zea mays*), common bean (*Phaseolus vulgaris)*, soybean (*Glycine max*), pearl millet (*Pennisetum glaucum*), finger millet (*Eleusine coracana*), and pea (*Pisum sativum*), which are well-known staple crops in African countries; the remaining publications deal with a further 24 plant species, and a few papers investigate more than one species. The list of all the investigated plant species with the related references is reported in Table 1.

To further restrict our analysis to research prominently conducted in Africa, only 48 publications were retained for further analysis, i.e., those with first and/or corresponding authors affiliated with an African country (Table 1, plant species and references in bold). Hence, 22 papers were excluded, as they either did not report an African affiliation as a prominent authorship [15,19,20,27,28,35,36,41,42,53,56,59,70,71,73,78,81,82,85], or the first author did not have an African affiliation, and there was no clear African research leadership due to the presence of at least three corresponding authors [43,60,69]. 

Egypt, Tunisia, Nigeria, Ghana, and South Africa are the most represented affiliated countries in these 48 publications (11, 8, 7, 5, and 5 publications each, respectively). Notably, the model plant *Arabidopsis thaliana* is not among the analyzed plants species (Table 1). 

A variety of journals with a broad range of impact factors (IFs) and publishers are represented in the 48-publication list, as detailed in Supplementary Table S1. Most important, three “research hotspots” emerged from content analysis of these 48 publications, i.e., (1) Fe deficiency and crops biofortification, (2) sustainable agriculture and fertilization with Fe nanoparticles, (3) Fe toxicity. These hotspots are described in detail in the following paragraphs.

## 3. Research Hotspot: Fe Deficiency and Crops Biofortification

Fe availability is low in alkaline soils; plants in arid and semiarid regions incur a shortage of Fe availability and hence, a Fe deficiency. Plants activate a complex array of morphological and biochemical responses to counteract Fe deficiency stress [86,87,88]. The isolation of plant genotypes from different crop species which are more tolerant to Fe deficiency represents a current effort pursued by several plant scientists in Africa. Such effort goes hand in hand with the search for and validation of Fe biofortification approaches. Also, efforts to achieve Fe enrichment in seeds is often associated with efforts to achieve Zn enrichment [29,84]. Surely, some hurdles have been identified in the various approaches, at least for cereals. The variability of the measured Fe content in seeds, as observed in various studies, could be due to the sensitivity of the adopted analytical method to improper post-harvest seed handling and also to data restricted to a single year [84]. The results obtained with rice, wheat, maize, barley, millet, and various legume species are detailed below; it is worth recalling that wild species have higher Fe content but lower yields than cultivated ones [3,84].

### 3.1. Rice

*Oryza glaberrima* is an African indigenous low-yielding rice species with various resistance traits, cultivated in West Africa for thousands of years [89,90]. Scientists from the West Africa Rice Development Association (WARDA) succeeded in producing fertile progenies from crosses of this species with *O. sativa* and further backcrosses with *O. sativa,* increasing stability and fertility for better performance in upland cultivation [89]. Later, hybrids from *O. sativa* × *O. glaberrima* crosses were produced for irrigated, lowland cultivation [90]. Some of these hybrids were named NERICAs (New Rice for Africa). Several concerns regarding the rigorous scientific assessments of the claims regarding NERICA rice as the “silver bullet” for Africa’s green revolution were raised [91]. Physical properties of NERICA varieties NERICA-1 and NERICA-4, Indica varieties IR-28 and IR-50, and Japonica variety Yumepirika were then compared; NERICA and Indica rice seeds had similar results in these tests [92]. A total of 445 NERICA × *O. sativa* rice lines have been field-tested in two different sites in Liberia during the wet season to identify QTLs for higher tolerance to Fe-toxicity; for that, four traits were scored: days to flowering, plant height, grain yield, and leaf bronzing score [21]. Also, 35 upland rice genotypes were grown in the field, in Ibadan, Nigeria, under Fe sufficiency or deficiency; three varieties tolerant to Fe deficiency were identified (FARO65, IRAT 109, NERICA3) [22].

### 3.2. Wheat

Foliar treatments with FeSO_4_ and/or ZnSO_4_ of winter wheat could alleviate drought symptoms [61], although the molecular mechanisms of interaction between Fe supplementations and such abiotic stress were not investigated. 

Various synthetic wheat lines were developed in the past by Japanese groups [93] by crossing the tetraploid wheat cultivar “Langdon” with *Aegilops tauschii* accessions mainly collected from Iran but also from other Asian countries; three lines with high Fe and Zn content in grains were identified; such traits were stable across the seasons [62]. Durum wheat genotypes tolerant to growth in calcareous soil were also selected [77]. 

### 3.3. Maize

Low nitrogen levels affect maize cultivation and yield in Sub-Saharan Africa (SSA), as well as the Fe and Zn content of seeds [30]. Several maize hybrids were grown in various experimental conditions (low or optimum N levels), and the most stable genotypes for Fe and Zn content of grains and for yield were identified under low N conditions [31]. Also, hybrid lines grown under low or optimum N conditions or under controlled drought stress were analyzed for genotype x environment interactions [32]. The authors reported a high positive correlation between grain Fe and Zn concentration (r = 0.97) and a moderate negative correlation between grain yield and Fe and Zn content (r = −0.43 and r = −0.44, respectively). The authors concluded that the development of Fe- and Zn-dense maize cultivars with high grain yield is feasible to combat Fe and Zn deficiency in SSA [32]. However, the effect of N treatments on total Fe grain content in maize is less clear, as reported in another study [33].

Efforts for multiple enrichments of maize with Fe, Zn, and also provitamin A have been conducted in Nigeria, with the identification of sets of hybrids which combined high Fe and Zn content (24.45 mg kg^−1^ and 29.24 mg kg^−1^) and average provitamin A content (7.48 mg kg^−1^); however, there was a weak but significant negative correlation between Zn and provitamin A, so the hybrids with the highest provitamin A content were also lowest in Zn content [34]. 

### 3.4. Barley

Barley (*Hordeum vulgare*) is another staple crop in North Africa and in Ethiopia, and a genome-wide association study (GWAS) of a collection of 496 spring barley genotypes (cultivars, improved lines, landraces) identified several single-nucleotide polymorphisms (SNPs) associated with Fe and Zn content, although several candidate genes are still annotated as “undescribed” [68]. 

### 3.5. Millet

Pearl millet is one of the six most important cereal crops for human nutrition in the world, and its relevance is increasing with global climate changes due to its resilience to high temperatures and drought [94]. Moreover, this so-called “nutricereal” has a very high nutritional value with respect to other cereals, and various breeding approaches for pearl millet are currently adopted [94]. Open-pollinated varieties (OPVs) of pearl millet were tested in various locations in West Africa for their growth performance and Fe and Zn grain content, with the identification of stable, high-Fe varieties [45,46,47]. Interestingly, quantitative genetics were applied to six generations of pearl millet obtained from two sets of parental lines grown at ICRISAT, Sadore, Niger, to model the trait inheritance of Fe grain content [48]. 

Finger millet also represents an important staple crop in various arid and marginal lands in eastern Africa, where other crops cannot thrive, as in Ethiopia, where finger millet represents one of the major staple foods [49]. Three different genotypes (Diga-01, Urji, Meba) grown in two different regions of Ethiopia and in two different slope configurations for each region were treated with Fe and Zn in a combination of the two micronutrients with the NPKS fertilization [49]. It emerged that, besides genotypes and treatments, the yield is dependent on location and slope, and all these variables should be considered for programs of agronomic biofortification [49].

Fertilization with phosphorus is beneficial to finger millet, causing an increase in calcium, Fe, and Zn content of grains from plants grown in three different locations in Kenya [50]. Again, the optimal level of phosphorus fertilizer is dependent on tested location [50]. This aspect should be considered to avoid the negative effects of unnecessary, supra-optimal concentrations of phosphorus for plants and soils.

### 3.6. Legumes 

Legumes show genetic variability in their tolerance to Fe deficiency; their inclusion in crop rotation improves soil nutritional status, thanks to symbiosis with N-fixing bacteria [83]. For these reasons, legumes are good candidates for sustainable agriculture [63,83]. As most Tunisian soils are calcareous, several Tunisian researchers studied Fe deficiency responses in different legume species in order to identify tolerant lines. Twenty genotypes of barrel medic (*Medicago truncatula*), a small annual legume used as forage but also as a model plant, were screened for their physiological response to Fe deficiency during growth. Fe deficiency-tolerant and -sensitive genotypes were identified; tolerance was correlated with greater acidification capacity, modified root architecture, and an induction of Superoxide Dismutase (SOD) activities [63,64]. Similar approaches were applied to investigate genotypic differences in the response to Fe deficiency in pea [51,52] and in common bean [37]. A set of 99 different cultivars and landraces of common bean were tested in different locations in Tanzania for their adaptability and stability [38]. Moreover, SNPs associated with grain Fe and Zn concentration were identified for such species [39]. In another study, Fe and Zn content of broad bean (*Vicia faba*) in Ethiopia was influenced by environmental conditions, especially soil properties [66].

## 4. Research Hotspot: Sustainable Agriculture and Fe Nanoparticles 

Soil fertilization with macro- and microelements is a common agricultural practice which needs to be tailored to the specific crop to limit leakage and soil degradation. The use of nanoparticles (NPs) is a practice explored in recent years; such research has been prompted by the evidence that parts of fertilizers (whether in soil or directly applied to plants) are not used by plants, and they are therefore wasted, with economical as well as ecological consequences. The production of micronutrient NPs would offer a sustainable alternative to traditional fertilizers due to their higher efficiency and reduced contamination. All these aspects, as well as the ways in which they are internalized by plants’ leaves and roots, the potential risks associated with their use (human health and food safety), and the need of rigorous assessments, have been recently reviewed [95,96]. 

A study described the green production of Fe-NPs, starting from extract of African wormwood (*Artemisia afra*) [72]. The authors characterized the physical and chemical properties of the obtained NP, thus demonstrating that they are genuinely formed by Fe oxides of 10–20 nm in diameter. These Fe-NPs influence the germination rate of both spinach and carrot seeds in a concentration-dependent way, and they can therefore act as nano-priming agents [72]. This, in turn, opens the question regarding the mechanisms by which Fe-NPs can promote seed germination in a given concentration range [72]. Another study compared the effects of conventional Fe treatment (ferric sulfate or Fe-EDDHA) with Fe-NPs on the composition in essential oils (EOs) of rose-scented geranium (*Pelargonium graveolens*). This perennial plant is cultivated worldwide, for instance in Egypt, and it is used for cosmetic and perfume production; its EO composition is dependent on nutritional status, and treating plants with Fe-NPs and humic acid boosts EO production in a superior way with respect to the other Fe treatments [75]. Also, the effects of treatment of orange trees (*Citrus sinensis*) grown in Egypt with green Fe-NPs (produced from fresh leaves of *Psidium guiaiava*, commonly known as guava) were evaluated in comparison with other conventional Fe treatments (FeSO_4_, Fe-EDTA) [79]. The appearance of produced Fe-NPs of needle-like shape at SEM seems different from the green Fe-NPs characterized in [72], and their chemical composition was not investigated; nonetheless, the improvement in most of the analyzed parameters in terms of physical and chemical features of fruits (yields, shelf life, mineral contents of fruits, etc.) would encourage the use of such Fe-NPs with respect to the other treatments for orange cultivation in arid regions [79]. Effects of foliar application of Fe-NPs, FeSO_4_, and Fe-chelate were also evaluated in broad bean grown in sandy soil, and Fe-NP gave superior results in terms of plant growth parameters and harvest index [67]. The superiority of Fe-NP fertilization with respect to conventional Fe or chelated Fe was also demonstrated in tomato for growth and yield [54].

Fe-NPs were also effective in ameliorating growth parameters in soybean [44]; commercial Fe-NPs (n-Fe_2_O_3_) were also effective in ameliorating growth parameters in sorghum seedlings, as well as their tolerance to salinity stress [57].

Sesame (*Sesamum indicum*) is produced in many African countries. An Egyptian group [76] studied the effect of treating three different sesame genotypes with a mixture of Fe, Mn, and Zn in nano form; however, the production of such Fe, Zn, and Mn fertilizers in the nano form has not been clearly described by authors.

Although these studies are promising and in line with the worldwide interest in precision agricultural practices, benefits observed from use of Fe-NPs have been not always confirmed, and indeed, in some cases, treatment with classic Fe-EDTA was more beneficial than with Fe-NPs, as described for sorghum plants [58]. 

Within the above cited results on this African research hotspot, we believe that discussion on health risks for exposed workers and on food safety for consumers due to exposure to Fe-NP via inhalation or via ingestion is still missing. Such risks should not be underestimated, as several studies reported endotheliar disfunction and inflammation, with increased pulmonary, vascular, and cardiac diseases, due to exposure to Fe-NPs of various diameters [97,98]. 

## 5. Research Hotspot: Fe Toxicity 

Fe toxicity is less frequent than Fe deficiency due to Fe chemistry; indeed, although Fe is abundant in the Earth’s crust, it is usually in highly insoluble Fe (III) oxidized form. Still, occasionally, Fe solubility can massively increase due to Fe reduction to Fe (II) form. This increase in Fe (II) availability can occur in anoxic conditions, such as in the so-called “rainfed lowlands on Fe-rich soils”, i.e., in Fe-rich soils exposed to prolonged floods and without satisfactory drainage ([23,99], or in acid sulfate soils, acid clay soils, and peat soils [100]. In all these conditions, Fe can be taken up in excess by root cells and exert its toxicity, with a consequent reduction in crop yields. The typical phenotypic hallmark of Fe toxicity is a brownish-red color, known as “bronzing”. 

Four plant defense mechanisms against Fe toxicity are adopted by plants [19,24]: (1) Fe exclusion, achieved by oxidation of Fe (II) into Fe (III) via oxygen released by roots; (2) Fe retention in metabolically inactive forms in cell vacuoles and within the Fe storage protein ferritin, mainly in plastids; (3) Fe partitioning in older plant tissues; and (4) ROS detoxification to alleviate oxidative load. Rice genotypes are broadly classified as Fe “excluders” or “includers”, relative to the adopted strategy [24]. 

Rice is the only major crop species which can incur Fe toxicity [99]; rice cultivations in several African regions are potentially exposed to Fe-toxicity conditions [23,24].

Praiseworthily, van Oort [23] mapped the Fe-toxicity spots in the African regions for the first time, together with maps of spots for other abiotic stresses (drought, cold, salinity, and sodicity). The various countries with the largest areas potentially at risk of Fe toxicity were identified, with Nigeria being at the highest risk. Also, a list of five countries with the largest surfaces cultivated with rice at risk of Fe toxicity was provided; such values were expressed as “rainfed lowland rice areas on Fe rich soil”/“total rice area”, and Togo was at the top of this list [23]. A predominance of West African countries is exposed to Fe toxicity, involving 12% of total rice area, thus representing a relevant threat for rice production. This percentage might become even larger, as further expansion of crop areas, which is expected in Africa for rice as well as for other cereals, could involve wetlands, which are currently neglected [23]. 

Tolerance mechanisms were investigated in seven different rice genotypes (KA-28, Bahia, Ciherang, IR64, L-43, Tsipala, X265), either tolerant of or sensitive to Fe toxicity, which were grown in the central highlands of Madagascar. Fe uptake rate, growth rate, and Fe partitioning between shoot tissues during vegetative and reproductive growth were measured, together with grain yield. Exclusion mechanisms, even in “excluder” genotypes, were relaxed during reproductive growth, indicating that mechanisms activated by plants to counteract Fe toxicity are dependent on developmental stage [24]. Strikingly, no correlation between grain yield and visual symptoms has been observed, indicating that the selection of tolerant lines based on visual symptoms is a simplistic approach [24]. These findings, together with the environmental dependence of intensity and the dynamics of Fe toxicity, highlight the need to unveil the genetic factors orchestrating responses against Fe toxicity. An RNA-seq analysis was performed to investigate the effects of magnesium (Mg) on Fe toxicity in the central highlands of Madagascar, as well as in hydroponic conditions, thus uncovering various genes potentially affecting the enhancement of tolerance by Mg [25]. Another research group analyzed Fe toxicity-tolerant rice varieties CK801 and Suakoko8, as well as sensitive varieties IR64 and Supa, under hydroponic conditions for their morphological and physiological responses to Fe toxicity [17,18]. In these studies, tolerant lines showed more lateral roots, a better development of aerenchyma, and higher O_2_ release. Hundreds of African rice *Oryza glaberrima* accessions were grown in the Plateaux region of Togo and analyzed for growth parameters, together with symptoms of Fe toxicity [26]. Physiological and biochemical responses against Fe toxicity were also studied in 15 accessions of cowpea (*Vigna unguiculata*) [65].

## 6. Other Research Lines

Analysis of fenugreek (*Trigonella foenum-grecum*) under increasing concentrations of Fe in the form of FeSO_4_ would encourage the use of such plants in Tunisian phytoremediation programs [74]. Roselle (*Hibiscus sabdaiffa* L.), known as karkadeh, is cultivated in various African regions, and it is a valuable commercial plant, as its sepals are used for human nutrition as well as for beauty cosmetic industries. Roselle plants grown in-field were sprayed with different compounds (Fe-EDTA, arginine, hemin, and their combinations), and the yield and chemical and elemental composition of their sepals were analyzed, showing stimulatory effects on flavonoid pigments [80].

## 7. Discussion

The responses of crops to Fe deficiency, including various approaches of plant biofortification with Fe, whether via fertilization or via genetic approaches, is a well-represented research topic in Africa; several lines of wheat, maize, rice, and various legumes have been examined to identify those of superior quality. Such research, in Africa, also involves millets; this is promising, since these species can better withstand adverse growth conditions and have the potential to also become an important crop outside Africa. Notably, the Food and Agriculture Organization of the United Nations (FAO) declared 2023 the International Year of Millets (IYM 2023) at https://www.fao.org/millets-2023/en (accessed on 14 September 2023) Hoboken, NJ, USA. The screening for Fe-dense millets is therefore of great importance [94]. Still, the molecular basis of Fe nutrition and homeostasis in millet is not known in the same detail as in other plant species. The international expansion of millet as a model plant in the plant Fe research community could therefore contribute to boosting its diffusion as a “nutricereal”.

Sustainable agriculture is becoming a growing concern not only in Africa but worldwide, requiring the adoption of eco-friendly cultivation practices for correct soil management and for the optimization of costs versus crop yield. Several scientists in the African continent are involved in the analysis of the effects of Fe-NP, indicating that this subject is very relevant for such parts of the world.

**Figure 1:**
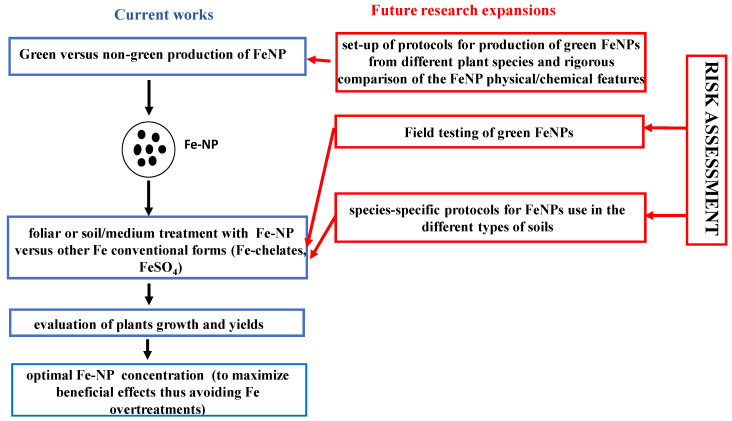
Current works and approaches on Fe nanoparticles (Fe-NPs) in the research conducted in the time span from 2018 to 2023 by prominently African-affiliated scientists (text boxes in blue, on the left) and the future research expansions suggested in the present work (text boxes in red, on the right). Current works: green and non-green Fe-NPs, produced with or without plant extracts, respectively; soil, growth medium, or foliar treatments with various concentrations of Fe-NP, Fe-chelates, or other conventional Fe treatments (e.g., FeSO_4_) have been performed on different plant species. To identify the best Fe-NP concentration to avoid overtreatments, plant growth and yield have been assessed. Future research expansions: a higher number of plant species could be tested as starting green extracts for the production of green Fe-NP; the physical/chemical features of the produced Fe-NP should be rigorously assessed. To optimize Fe-NP use in the environment, protocols specifically adapted for optimal treatment of each plant species with green Fe-NP should be established and shared within the scientific community. A rigorous risk assessment is needed: direct effects on human health during Fe-NP treatments and indirect effects on human health in terms of food and soil safety should be considered.

Several approaches can be followed to synthesize Fe-NP, and one of them is based on a bottom-up approach in which NPs can grow from reaction precursors by using plant extracts rich in organic reductants [101]. Several plant extracts have already been used for such synthesis, and various mechanisms have been proposed for the reactions involved [101]. Still, the establishment of species- and developmental stage-specific protocols for Fe-NP preparation and use in terms of concentration and times of treatments is still needed. These shared protocols should also clearly include the green Fe-NP preparations. A systematic comparison of green Fe-NP versus Fe-NP prepared with other chemical methods would help disentangle the variety of results which are emerging from various research groups; this, in turn, would offer shared avenues of collaborative approaches to this relevant research field among scientists from different countries and with different expertise (Figure 1). Moreover, in this identified research hotspot, we noticed a general lack of risk assessment of Fe-NPs in terms of human health upon exposure to Fe-NPs during handling, but also in terms of food safety. Fe-NP risk assessment is for sure a compulsory step in any future research involving use of nanomaterials in agriculture (Figure 1). Historically, NP emissions from industrial or traffic sources have been associated with health risks; however, the expansion in the use of Fe-NPs for novel agricultural approaches requires a rigorous risk assessment of the nanomaterials within this application field, for human health [97,98] but also for the whole environment [102]. Notably, proposals to modify surfaces of nanomaterials to make them more biocompatible and reduce the inflammatory/oxidative responses of human tissues have been put forward [97]; such hazard reduction strategies have been named “safe by design” [103]. 

**Figure 2:**
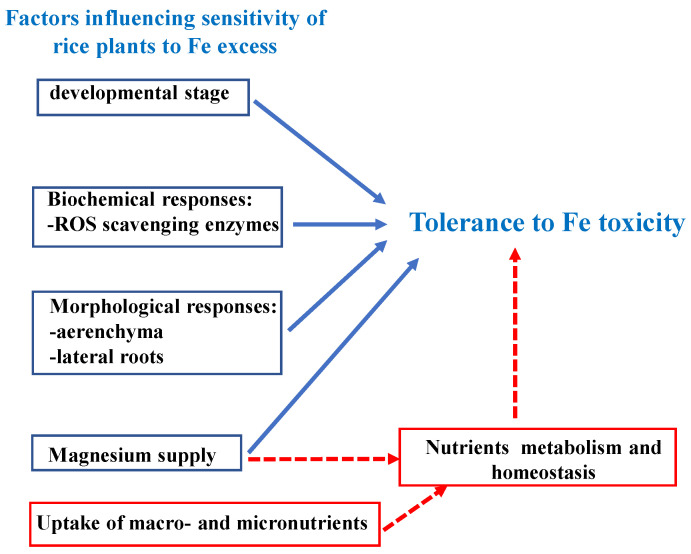
Current findings on tolerance/sensitivity to Fe toxicity in rice in the research conducted in the time span from 2018 to 2023 by prominently African-affiliated scientists (blue boxes) and the research potentialities suggested in the present work (red boxes). The sensitivity of rice plants to Fe excess is dependent on anatomical traits (extent of root aerenchyma and expansion of lateral roots), on the involvement of the ROS scavenging enzymes, and on Mg nutritional status of the plant; also, responses to Fe excess are dependent on developmental stage. All these features contribute to tolerance/sensitivity of rice plants to Fe toxicity. Possible future research potentialities could involve the analysis of the uptake processes and of the nutritional status of the other essential nutrients under Fe excess, together with the analysis of their homeostatic adjustments.

Research on Fe toxicity is also well represented in African countries, as it is driven by the need to ameliorate crop yield and quality of lowland rice. The extent of African soil prone to Fe toxicity and the increasing African population and consequent increasing need of crop production, including rice, make the understanding of Fe toxicity of great importance for millions of people. Some molecular aspects of plant exposure to Fe excess have been analyzed in the past in model and/or crop plants, such as the regulatory mechanisms of Fe sequestration through the iron storage protein ferritin [104,105,106,107,108,109]. Also, below-ground as well as above-ground adaptive mechanisms of rice to Fe toxicity have been elucidated [19,100]. Still, various aspects of plant response to Fe excess and defense mechanisms against Fe toxicity need to be disentangled. Fe toxicity is indeed often accompanied by deficiencies in other nutrients, such as potassium, magnesium, calcium, and silicon [19]. The impact of such deficiencies in the development of Fe toxicity symptoms during exposure to Fe excess and their homeostatic adjustments from root to seed in tolerant versus Fe-sensitive species might contribute to the identification of markers of tolerance for breeding programs (Figure 2). Most of the gene regulators involved in the responses against Fe toxicity are also unknown [100].

## 8. Conclusions and Future Directions

Predictions on climate change and growth of human population make plant Fe science of utmost relevance; more accurate working models on Fe nutrition and metabolism in plants are needed for crops of established economic and nutritional value, as well as for plants with emerging nutritional, economic, and ecological importance. Hence, any relevant advancement in this field should be worthy of consideration, and its dissemination within the scientific community should be favored. The present work therefore turns the spotlight on plant Fe research mainly conducted in Africa, with an overview of the current research situation. Three main research hotspots were identified, and future research expansions were proposed, hopefully fostering initiatives with research groups worldwide.

The 48 research products identified in the time span from 2018 to 2023 are affiliated with 22 countries; hence, more than half of the African countries are either not involved in any research on plant Fe or did not succeed in publishing any Scopus-indexed work. Also, around one fifth of such research products have been published in minor journals without an impact factor. This analysis reveals that an expansion of African plant Fe research, both quantitative and qualitative, is welcome, as it could be beneficial for Africa itself, but also outside this continent. For example, a similar Scopus search on plant Fe research conducted on Southeast Asian territories highlighted a limited number of total publications (22), with Thailand, Indonesia, the Philippines, and Vietnam as affiliated countries; interestingly, half of the publications were related to plant response against Fe toxicity (not shown). Hence, Fe toxicity is an economical and nutritional burden for many low-income countries worldwide. Last, ongoing projects of Fe research in Africa, if unpublished, have not been included in the present review to avoid any bias regarding retrieval, visibility, sponsorship, or funding of unpublished results. We nonetheless hope that the present work can be handled as a useful tool by plant Fe scientists worldwide to come in direct contact with Fe research conducted in Africa, as well as for future collaborations and shared initiatives. Scholarships and shared organizations of scientific sessions at congresses in plant physiology and nutrition might represent starting points for such collaborative initiatives. 

## Figures and Tables

**Table 1:** List of investigated species in publications on plant Fe science and affiliated with African countries, from 2018 to 2023, as retrieved by the Scopus search described in the text. Columns from left: plant species, references of publications referring to the species, and publications with prominent African affiliations. Species investigated in publications with prominent African affiliations are in bold.

Plant Species	Publications	Publications with African Prominent Authorship
**rice** **(various *Oryza* species)**	[17,18,19,20,21,22,23,24,25,26]	[17,18,21,22,23,24,25,26]
**maize** **(*Zea mays*)**	[27,28,29,30,31,32,33,34]	[29,30,31,32,33,34]
**common bean** **(*Phaseolus vulgaris*)**	[35,36,37,38,39,40]	[37,38,39,40]
**soybean** **(*Glycine max*)**	[27,41,42,43,44]	[44]
**pearl millet** **(*Pennisetum glaucum*)**	[45,46,47,48]	[45,46,47,48]
**finger millet** **(*Eleusine coracana*)**	[33,49,50]	[33,49,50]
**pea** **(*Pisum sativum*)**	[51,52]	[51,52]
**tomato** **(*Solanum lycopersicum*)**	[53,54,55]	[53,54,55]
**sorghum** **(*Sorghum bicolor*)**	[56,57,58]	[57,58]
lentil (*Lens culinaris*)	[59,60]	
**wheat** **(various species)**	[61,62]	[61,62]
**barrel medic** **(*Medicago truncatula*)**	[63,64]	[63,64]
**cowpea** **(*Vigna unguiculata*)**	[33,65]	[33,65]
**broad bean** **(*Vicia faba*)**	[66,67]	[66,67]
**barley** **(*Hordeum vulgare*)**	[68]	[68]
flax (*Linum usitatissimum*)	[69]	
date palm (*Phoenix dactylifera*)	[70]	
grey mangrove (*Avicennia marina*)	[71]	
**African wormwood** **(*Artemisia afra*)**	[72]	[72]
**spinach** **(*Spinacia oleracea*)**	[72]	[72]
**carrot** **(*Dacus carota*)**	[72]	[72]
Chinese mandarine (*Citrus reticulata* Blanco)	[73]	
**fenugreek** **(*Trigonella foenum-graecum*)**	[74]	[74]
**rose-scented geranium (*Pelargonium graveolens*)**	[75]	[75]
**sesame** **(*Sesamum indicum*)**	[76]	[76]
**durum wheat** **(*Triticum durum*)**	[77]	[77]
alfalfa (*Medicago sativa*)	[78]	
Washington navel orange (*Citrus sinensis*)	[79]	
**roselle** **(*Hibiscus sabdariffa*)**	[80]	[80]
grape (*Vitis vinifera*)	[81]	
Sulla carnosa (*Hedysarum carnosum*)	[82]	
**legumes**	[83]	[83]
**cereals**	[84]	[84]

## Data Availability

The data presented in this study are available in this article and in its Supplementary Materials.

## Supplementary Materials

The following supporting information can be downloaded at https://www.mdpi.com/article/10.3390/plants13010014/s1. Figure S1: Keywords excluded from Scopus search; Figure S2: Journals excluded from Scopus search; Figure S3: Affiliated African countries in the 48 publications on plant science prominently produced by African research groups in the time span from 2018 to 2023; Table S1: List of journals in which the 48 publications of plant Fe science with African affiliations in a prominent position have been published, from 2018 to 2023. For each journal, impact factor IF and the number of publications published in that journal are given. Lack of IF is indicated as “not available” (n.a.).
