# Supplementary material for: Plant Iron Research in African Countries: Current “Hot Spots”, Approaches, and Potentialities
Source: Plants (Basel). 2023 Dec 19;13(1):14. doi: 10.3390/plants13010014 (PMC10780554; doi:10.3390/plants13010014)
Supplement: Supplementary file 1 [file plants-13-00014-s001.zip › plants-2748363-supplementary.pdf]

"Nonhuman", "Human", "Controlled Study", "Humans", "Animal", "Animals", "Priority Journal", "Unclassified Drug", "Male", "Female", "Animal Experiment", "Mouse", "Drug Effect", "Physiology", "Human Cell", "Adult","Animal Tissue","Mice", "Animal Model", "Animal Cell", "Hepcidin", "Pathology", "Review" "Catalysis", "Hemoglobin", "Nanoparticles", "Rat", "Blood", "Rats", "Iron Blood Level", "Heme", "Major Clinical Study", "Hepcidins", "Concentration (composition)", "Soils", "Bacterium", "Bacteria", "Erratum" "China", "Biogeochemistry", "Particle Size", "Experimental Study", "Cyanobacteria", "Groundwater", "Hematite", "Pollutant Removal", "Dissolution", "Iron Plaque", "Wetlands", "Animal Compost", "Bioaccessibility", "Biodegradation, Environmental", "Food Security", "3% NaCl", "Acrylic Resins", "Acrylic Acid Resin", "Bearing Wastes", "Atmospheric Thermodynamics", "Atmospheric Chemistry", "Atmospheric Aerosols", "Article" "Aquatic Organisms", "Aquaponics", "Population Structure", "Anionic Dye","Anionic Dyes", "Anionic And Cationic Dyes", "Binary System", "Binary Systems""Bouake","Buchwald-Hartwig", "Clinker Ash", "Classification", "Continuum Removal","Cow Manure".

Figure Supplementary S1

List of keywords excluded from Scopus search

"Chemistry an Asian Journal", "Lwt", "Food Science and Technology Brazil", "Acta Pedologica Sinica", "Food Chemistry", "Frontiers in Sustainable Food Systems", "Journal of Food Science and Technology","Iron Catalysis Design and Applications", "Isme Journal", "Fermentation", "Food and Function", "Food Research International", "Journal of Applied Phycology", "Journal of Fluorescence", "Animal Feed Science and Technology", "Aquaculture", "Geoderma", "European Journal of Soil Science", "Journal of Phycology", "Journal of The American Society of Brewing Chemists", "Brazilian Journal of Food Technology", "Food Control", "Iranian Journal of Applied Animal Science", "Journal of Food Science", "Luminescence", "Shipin Kexue Food Science", "Trends in Food Science and Technology", "ACS Food Science and Technology", "Algal Research", "Aquatic Sciences", "Food Science and Nutrition", "Soil and Sediment Contamination","International Food Research Journal", "International Journal of Food Sciences And Nutrition", "Journal of Clinical and Diagnostic Research", "Journal of Culinary Science And Technology", "Journal of Entomological Research", "Journal of Food Composition and Analysis", "Poultry Science", "Revista Brasileira De Ciencia Avicola Brazilian Journal of Poultry Science", "Studia Universitatis Vasile Goldis Arad Seria Stiintele Vietii", "Biointerface Research in Applied Chemistry", "Bioinorganic Chemistry and Applications", "International Journal of Food Science", "Letters in Applied Nanobioscience".

Figure Supplementary S2

List of Journals excluded from Scopus search

“Egypt, "South Africa", "Tunisia", "Nigeria", "Morocco", "Ethiopia", "Ghana", "Uganda", "Kenya", "Algeria","Cote d'Ivoire", "Zimbabwe", "Burkina Faso", "Cameroon", "Madagascar", "Senegal", "Tanzania", "Benin", "Niger", "Sudan", "Botswana", "Liberia", "Libyan Arab Jamahiriya", "Mauritania", "Sierra Leone", "Togo"

Figure Supplementary S3

Affiliated African Countries in prominent position, in the 48 publications on plant Fe science, in the time span 2018-2023.

**Journal name**	**IF**	**nr. publications**
Agronomy (MDPI)	3.7	7
Plants (MPDI)	4.5	5
Frontiers in Plant Science (Frontiers)	5.6	4
Field Crops Research (Elsevier)	5.8	2
Crop and Pasture Science (Csiro Publishing)	1.9	2
Australian Journal of Crop Science (Southern Cross Publishing)	**n.a.**	2
Journal of Plant Nutrition (Taylor & Francis)	2.1	2
Functional Plant Biology (Csiro Publishing)	3.0	2
Plant Breeding (Wiley)	2.0	1
Phyton-International Journal of Experimental Botany (Tech Science Press)	1.7	1
ACS-Agricultural Science and Technology (ACS Publications)	2.5	1
Cereal Research Communications (Springer)	1.6	1
Journal of Crop Science and Biotechnology (Springer)	**n.a.**	1
Plant and Soil (Springer)	4.9	1
Journal of Soil Science and Plant Nutrition (Springer)	3.9	1
Plant Direct (Wiley)	3.0	1
International Journal of Agronomy (Hindawi)	1.9	1
International Journal of Vegetable Science (Taylor & Francis)	**n.a.**	1
Plant Cell Biotechnology and Molecular Biology (I. K. Press)	**n.a.**	1
Journal of Cereal Science (Elsevier)	3.8	1
Ecological Genetics and Genomics (Elsevier)	1.9	1
Agricultural science digest (ARCC Journals)	**n.a.**	1
Asian Journal of Plant Science (ANSInet)	**n.a.**	1
Acta Agriculturae Slovenica (University of Ljubljana Press)	**n.a.**	1

Pakistan Journal of Biological Sciences (ANSInet)	**n.a.**	1
Plant Species Biology (Wiley)	1.4	1
Journal of Agronomy and Crop Science (Wiley)	3.5	1
Crop Science (Crop Science Society of America)	2.3	1
Plant Genetic Resources: Characterization and utilization (Cambridge University Press)	1.1	1
Iraqi Journal of Agricultural Sciences (University of Baghdad)	**n.a.**	1

Table Supplementary S1

List of Journals in which the 48 publications of plant Fe science with African affiliation in the prominent position, from 2018 to 2023, have been published. For each Journal, Impact Factor IF and the number of publications published in that Journal are given. Lack of IF is indicated as "not available" (n.a.).
